# The Effects of Muscle Cell Aging on Myogenesis

**DOI:** 10.3390/ijms22073721

**Published:** 2021-04-02

**Authors:** Athanasios Moustogiannis, Anastassios Philippou, Orjona Taso, Evangelos Zevolis, Maria Pappa, Antonios Chatzigeorgiou, Michael Koutsilieris

**Affiliations:** 1 Department of Physiology, Medical School, National and Kapodistrian University of Athens, 75 MicrasAsias, 115 27 Goudi-Athens, Greece; tfilipou@med.uoa.gr (A.P.); o.taso@ucl.ac.uk (O.T.); ezevolis@med.uoa.gr (E.Z.); achatzig@med.uoa.gr (A.C.); mkoutsil@med.uoa.gr (M.K.); 2 First Department of Propaedeutic Internal Medicine, Joint Rheumatology Program, National and Kapodistrian University of Athens, 75 MicrasAsias, 115 27 Goudi-Athens, Greece; mariak.pappa@yahoo.com

**Keywords:** myoblasts, myogenesis, cellular senescence, aging, sarcopenia, muscle atrophy

## Abstract

The process of myogenesis gradually deteriorates as the skeletal muscle ages, contributing to muscle mass loss. The aim of this study is to investigate the effect of senescence/aging on skeletal myogenesis, in vitro. A model of multiple cell divisions of C2C12 myoblasts was used to replicate cell senescence. Control and aged myoblasts were investigated during myogenesis, i.e., at days 0, 2, and 6of differentiation. SA-β-gal activity and comet assay were used as markers of aging and DNA damage. Flow cytometry was performed to characterize potential differences in cell cycle between control and aged cells. Alterations in the mRNA and/or protein expression of myogenic regulatory factors (MRFs), IGF-1 isoforms, apoptotic, atrophy, inflammatory, metabolic and aging-related factors were evaluated. Compared with the control cells, aged myoblasts exhibited G0/G1 cell cycle arrest, DNA damage, increased SA-β-gal activity, and increased expression of aging-related factors p16 and p21 during differentiation. Moreover, aged myoblasts showed a reduction in the expression of MRFs and metabolic/anabolic factors, along with an increased expression of apoptotic, atrophy and inflammatory factors. A diminished differentiation capacity characterized the aged myoblasts which, in combination with the induction of apoptotic and atrophy factors, indicated a disrupted myogenic lineage in the senescent muscle cells.

## 1. Introduction

On 1931, MacDonald Critchley was the first to recognize that skeletal muscle mass decreases with age. Indeed, one of the most serious consequences of aging is the progressive loss of muscle mass (sarcopenia) and function, which affects the quality of life and, ultimately, the survival of elderly people [1,2]. A negative protein turnover [3], impaired mitochondrial dynamics [4], decreased muscle regeneration capacity [5,6], decreased production of anabolic factors [7], as well as an exacerbation of apoptosis are considered as cellular mechanisms involved in the progressive decline in muscle mass (atrophy) with age that lead to sarcopenia [8].

A central hallmark of aging is senescence, which refers either to the gradual deterioration of functional characteristics of the whole organism or to cellular senescence. Cellular senescence, first described by Hayflick and Moorhead in the 1960s, is characterized by the irreversible arrest of cells following prolonged cultivation [9,10]. Specifically, cellular senescence, or replicative cell senescence, was described as a progressive decrease in growth rate and concomitant increase in cell death [10]. The induction of cellular senescence is typically associated with increased expression of β-galactosidase (SA-β-gal) [11,12], elevate levels of DNA damage [13,14], upregulation of aging related proteins p16 [15,16,17], p21 [18,19], reduced proliferative and differentiation ability of myoblasts [20,21], and higher proportions of cells in G0/G1 cell cycle phase [22,23].

The C2C12 cell line is a murine myoblast cell line widely used as an in vitro model of skeletal muscle cell biology [24,25,26], while the myogenic differentiation of C2C12 myoblasts into myotubes represents a well-established model of muscle cell differentiation processes [27]. Specifically, C2C12 cells were originally derived from satellite cells from the thigh muscle of mice after a crush injury; thus, they represent the in vitro cell line of tissue satellite cells. The latter are myogenic cells activated in muscle regeneration and can proliferate, differentiate, fuse, and lead to new myofiber formation [28]. Similarly, C2C12 cells possess the ability to proliferate, differentiate, and fuse into visible mature myotubes. Myogenic differentiation is driven by multiple signal transduction pathways, which coordinate the balance between muscle growth and atrophy, or protein synthesis and protein degradation [29]. In this context, the myogenic regulatory factors (MRFs) are a group of four muscle-specific transcription factors, including myogenic factor 5 (Myf5), MyoD, myogenin, and myogenic regulatory factor4 (MRF4), whichact differentially during myogenesis to cooperatively regulate skeletal muscle growth and development [28,30].

In addition, a key anabolic factor that plays a central role in both the proliferation and myogenic differentiation of skeletal muscle cells is insulin-like growth factor 1 (IGF-1) [31,32,33,34,35], while potentially differential actions of its isoforms in skeletal muscle biology have been proposed [36,37,38]. On the other hand, muscle-specific atrophy factors along with pro-apoptotic factors are negative regulators of muscle growth and development [39]. Specifically, myostatin activity prevents muscle growth, inhibits myoblast differentiation and protein synthesis, reduces myotube size, and inhibits IGF-1-induced myotube hypertrophy [40,41]. Moreover, atrogin-1 (MaFbx) and muscle ringer finger-1 (MuRF-1) activation cause muscle protein degradation [42,43]. Furthermore, important pro-apoptotic factors, such as FoxO1 and p53, have been negatively implicated in muscle cell growth and differentiation, and when skeletal muscle cells undergo atrophy, a subset of myoblasts exhibits apoptosis [44]. In particular, overexpression of FoxO1 has been found to dramatically inhibit C2C12 myoblast differentiation [45], while many studies have revealed a close association between p53, muscle stem cell perturbation, and muscle aging, with the latter being characterized by muscle atrophy and a decline in regenerative capacity [46,47,48,49,50].

However, the cellular dysfunctions, and their links and interactions associated with senescence in skeletal muscles cells remain largely unknown. The purpose of this study was to further investigate the effect of cellular senescence on myogenic differentiation, in the context of characterizing cellular and molecular responses of aged muscle cells during myogenesis, using an in vitro model of myoblast aging. This work is part of a larger research project, in which the aged cells will be treated with various factors, agonists, and antagonists of specific pathways, as well as being mechanically loaded in vitro, in order to investigate the molecular mechanisms and signaling pathways, including those induced by mechanical loading, whichregulate the myogenic differentiation capacity of the aged myoblasts, ultimately drawing a mechanistic model for myoblast senescence-induced skeletal muscle alterations in aging.

## 2. Results

### 2.1. Cell Cycle Analysis

**Figure 1:**
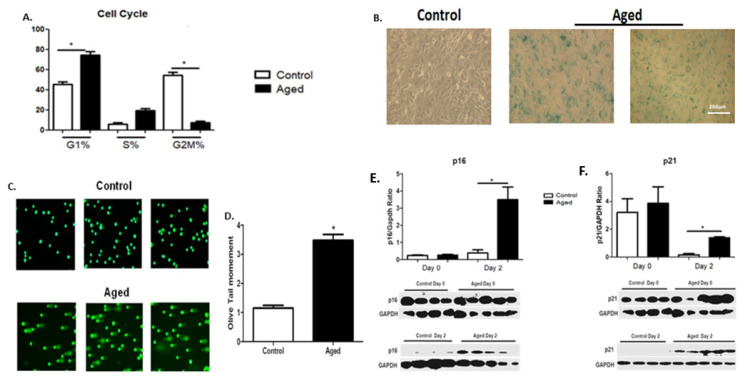
(**A**) Effects of myoblast senescence on cell cycle progression. Percentages of cells in each phase arerepresented for the control and aged myoblasts. In contrast to the controls, the aged myoblasts exhibited an aging phenotype with an arrest of cell cycle progression, i.e., increased number of cells (%) in the G1 phase along with a reduction incell number in the G2/M phase. (**B**) Increased SA-β-gal activity as a result of myoblast aging. Representative images of the increased SA-β-gal-positive blue-green stain cells in the aged myoblast cultures. (**C**) Increased DNA damage as a result of myoblast aging. Representative alkaline comet assay images of aged myoblasts and control cells. (**D**) Quantification of the endogenous DNA damage in aged myoblasts compared to controls. (**E**,**F**) Effects of myoblast aging on the expression of cellular senescence-associated proteins p16 and p21. Three independent experiments were performed, and 200 cells per sample were scored. Representative Western blots and immunoblotting quantification of p16 (**E**) and p21 (**F**) expression in aged myoblasts compared to controls in the second day of their differentiation process. The expressions of the proteins were normalized to each corresponding GAPDH on the same immunoblot (Mean ± SE of 3 independent experiments performed in triplicate; * *p* < 0.05).

To investigate the potential effects of cellular senescence on C2C12 myoblasts, cell cycle analysis was performed by flow cytometry in the control and aged cells. In contrast to the control myoblasts, a G1-phase arrest of cell cycle progression along with a reduction incell number (%) in the G2/M phase was found in the aged myoblasts (Figure 1A).

#### 2.1.1. Senescence-Associated β-Galactosidase (SA-β-gal) Staining

We examined SA-β-gal activity as a marker of cellular senescence in control and aged myoblasts. Compared to controls, aged myoblasts exhibited increased activity of SA-β-gal, suggesting increased lysosomal activity in those cells (Figure 1B).

#### 2.1.2. DNA Damage

Tail moments (TMs) which reflectedthe frequency of DNA breaks wereused to quantify DNA damage with alkaline comet assay in both the control and aged myoblasts. In aged myoblasts, DNA damage was higher than in the control myoblasts, as indicated by the increased TM levels in those cells (Figure 1C,D). This is a prominent feature of senescence, also compatible with the increased lysosomal activity (SA-β-gal) (Figure 1B) and the G0/G1 phase cell cycle arrest (Figure 1A) observed in the aged myoblasts.

#### 2.1.3. Expression of Cellular Senescence Markers during Myoblasts Differentiation

Along with the increased levels of SA-β-gal activity (Figure 1B), DNA damage (Figure 1C,D) and the cell cycle arrest in G0/G1 phase (Figure 1A), the aged myoblasts were characterized by the elevated expression of cellular senescence markers p16 (Figure 1E) and p21 (Figure 1F) in their early differentiation.

### 2.2. Effects of Myoblast Aging on the Expression of Myogenic Regulatory Factors (MRFs)

**Figure 2:**
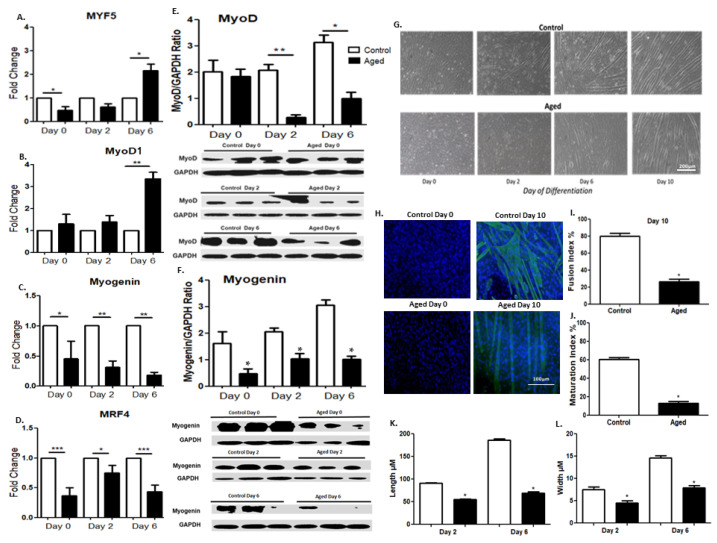
(**A**–**D**) Myogenic regulatory factor expression during myoblast differentiation. Quantitative analysis of the muscle-specific transcription factors MyF5 (**A**), MyoD (**B**), myogenin (**C**) and MRF4 (**D**) mRNA expression in aged myoblasts compared to controls during their differentiation processes. The mRNA expression values of MRFs in aged myoblasts have been normalized to the corresponding GAPDH mRNA and are expressed as fold changes compared to control cells. (**E**,**F**) Effects of myoblast aging on their expression of the myogenic regulatory factors MyoD and myogenin. Representative Western blots and immunoblotting quantification of MyoD (**E**) and myogenin (**F**) in aged myoblasts compared to control cells during their differentiation process. The values of the proteins of interest were normalized to each corresponding GAPDH on the same immunoblot. (**G**) Morphology-based analysis of control and aged C2C12 myoblasts during differentiation. Bright-field microscopy shows delayed myotubes formation in the aged myoblasts compared with the control cells over time, during myogenesis. (**H**) MyHC and DAPI immunostaining revealed a substantial reduction inmyotubes in aged cells. (**I**) Fusion index and (**J**) maturation index values were calculated in control and aged myotubes immunostained with MyHC. Myotubes were considered differentiated cells that contained more than three nuclei. The fusion index was defined as the percentage of nuclei present in myotubes over the total number of nuclei present in the observed field, while maturation index was defined as the percentage of nuclei present in myotubes that contained more than 10 nuclei over the total number of nuclei present in the observed field. (**K**,**L**) Morphological analysis of myotubes observed after 2 and 6 days of differentiation at both control and aged groups. Length and width of myotubes of each group have been measured and reported as mean values in the graph. Data wereselected from 10 different and randomly chosen microscopic fields. (Mean ± SE of 3 independent experiments performed in triplicate; * *p* < 0.05, ** *p* < 0.01, *** *p* <0.001).

To investigate the effects of myoblasts aging on their myogenic potential, we examined the protein and/or mRNA expression levels of both early (Myf5, MyoD) and late (myogenin, MRF4) differentiation factors in the aged C2C12 myoblasts during their differentiation into myotubes. As compared to the control cells, the aged myoblasts showed a late increased mRNA expression of the early differentiation factors Myf5 and MyoD on day sixof differentiation, while the late differentiation factors myogenin and MRF4 exhibited a reduced expression almost throughout the differentiation period, (Figure 2C,D). At the protein level, a reduced expression of MyoD and myogenin was documented during the differentiation of aged myoblasts compared to the controls, which is compatible with an overall delayed differentiation process in the aged myoblasts (Figure 2E,F). In particular, MyoD has been generally considered as an early differentiation myogenic factor, and its expression is expected to be downregulated at the late/terminal differentiation stage of myoblasts [51]. Nevertheless, due to the multiple biological roles of this key myogenic factor throughout the myogenic process [29], it has been reported to exhibit a steady state level throughout the progression of the myogenic differentiation [52], as is also shown in our experiments, without significant changes over time. Moreover, morphological differences were documented between the control and aged myotubes, corroborating a delayed differentiation (myotubes formation) process of the latter cells (Figure 2G). Specifically, the aged cells exhibited a reduced number of myotubespositively stained with the terminally differentiation marker myosin heavy chain (MyHC) (Figure 2H), as documented by the fusion index (FI) and the maturation index (MI) values, which were significantly lower in the aged cells compared to controls (FI: 23% vs. 79% and MI: 12% vs. 60%; Figure 2I,J, respectively). In addition, morphometric analysis of myotube size revealed that both the length and width of the aged myotubes were significantly smaller compared to the controls (Figure 2K,L).

### 2.3. Effects of Myoblast Aging on the Expression of Anabolic Factors during Myogenesis

**Figure 3:**
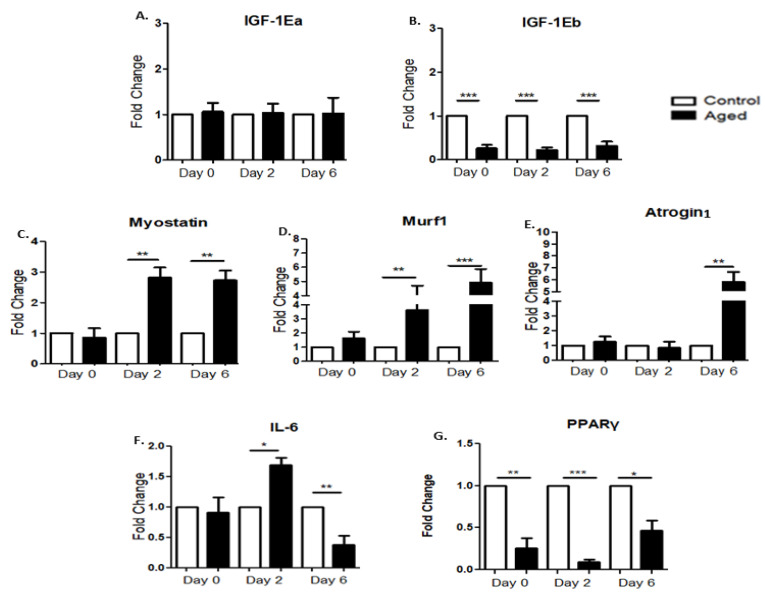
(**A**,**B**)Effects of myoblast aging on the expression of IGF-1 isoforms. Quantitative analysis of IGF-1Ea (**A**) and IGF-1Eb (**B**) mRNA expression in aged myoblasts compared to controls during their differentiation. (**C**–**E**) Effects of myoblast aging on the expression of muscle atrophy genes during myoblast differentiation. Quantitative analysis of myostatin (**C**), Murf1 (**D**) and Atrogin1 (**E**) mRNA expression in senescent myoblasts compared to control cells during their myogenic differentiation. (**F**,**G**) Effects of myoblast aging on the expression of inflammatory and metabolic factors. Quantitative analysis of IL-6 (**F**) and PPAR-γ (**G**) mRNA expression in senescent myoblasts compared to control cells during their differentiation. The mRNA values of the factors of interest in aged myoblasts have been normalized to the corresponding GAPDH mRNA and are expressed as fold changes compared to control myoblasts. (Mean ± SE of 3 independent experiments performed in triplicate; * *p* < 0.05, ** *p* < 0.01, *** *p* < 0.001).

IGF-1 is a major regulator of skeletal muscle development and growth that can induce hypertrophy and block atrophy; therefore, we examined the effects of myoblast aging on the expression profile of IGF-1 isoforms during the myogenic differentiation of myoblasts. We did not find any significant differences between the control and aged myoblasts regarding the expression of the IGF-1Ea isoform during myogenesis (Figure 3A). However, an impaired response was revealed regarding the other isoform, IGF-1Eb, exhibiting a reduced expression in the aged myoblasts compared with the control cells during the first days of differentiation (Figure 3B).

### 2.4. Effects of Myoblast Aging on the Expression of Muscle Atrophy Factors during Myogenic Differentiation

We also examined the effects of myoblast aging on the expression of muscle atrophy genes during the differentiation process. It is notablethat in contrast to the anabolic factor, IGF-1Eb decreased expression, and the aged myoblasts exhibited significant increases in the expression of the atrophy genes myostatin, Murf1 and Atrogin1 as compared to controls during myogenic differentiation (Figure 3C–E).

### 2.5. Effects of Myoblast Aging on the Expression of Inflammatory and Metabolic Factors during Myogenesis

We further examined the expression of a key metabolic factor, PPAR-γ, during the myogenic differentiation process, the activation of which in myocytes induces a local production of adiponectin that acts on muscle cells to improve their insulin sensitivity [53]. In addition, weinvestigated the expression of IL-6,a main factor modulating the complex relationship between aging and chronic morbidity [54]. Compared to the control myoblasts, aged cells exhibited a significant upregulation of IL-6 on the secondday of differentiation followed by its downregulation on the sixthday (Figure 3F). Interestingly, the aged myoblasts showed reduced expression levels of PPAR-γ compared to control cells throughout their myogenic differentiation (Figure 3G).

### 2.6. Effects of Myoblast Aging on the Expression of Pro-Apoptotic Factors

**Figure 4:**
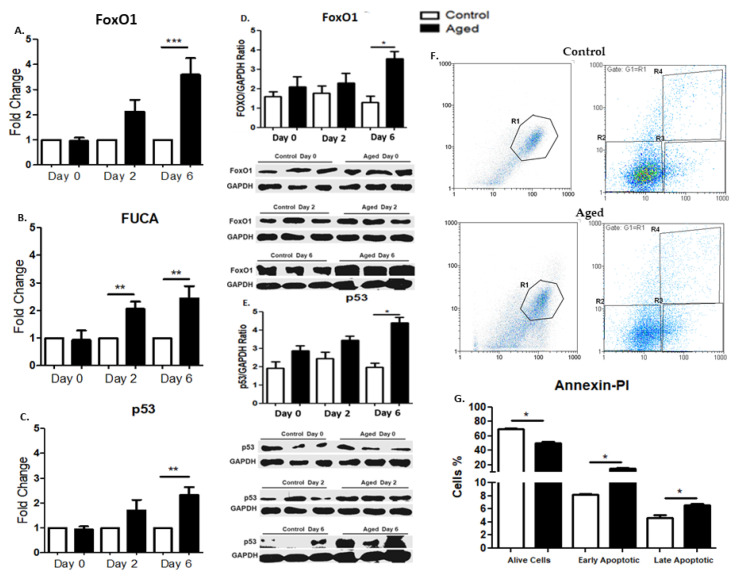
(**A**–**C**) Effects of myoblast aging on the expression of pro-apoptotic genes during myogenic differentiation. Quantitative analysis of FoxO1 (**A**), FUCA (**B**), and p53 (**C**) mRNA expression in aged myoblasts compared to controls during their differentiation. The mRNA expression values of pro-apoptotic factors in the aged myoblasts have been normalized to the corresponding GAPDH mRNA and are expressed as fold changes compared to control myoblasts. (**D**,**E**) Effects of myoblast aging on the expression of pro-apoptotic proteins FoxO1 and p53.Representative Western blots and immunoblotting quantification of FoxO1 (**D**) and p53 (**E**) in aged myoblasts compared to control cells during their myogenic differentiation. The values of the apoptotic proteins were normalized to each corresponding GAPDH on the same immunoblot. (**F**,**G**) Effects of myoblast aging on cell death (Annexin-PI). (**F**) Histograms from a representative experiment show the apoptotic effect of senescence on myoblasts. The percentages of necrotic, live, early apoptotic, and late apoptotic cells are displayed in R1, R2, R3 and R4, respectively. (**G**) Bar graphs show that senescence induced the apoptosis of (aged) myoblasts. Quantitative results (R2–R4) are displayed aspercentagechanges compared to the control. (Mean ± SE of 3 independent experiments performed in triplicate; * *p* < 0.05, ** *p* < 0.01, *** *p* < 0.001).

Along with the muscle atrophy genes, we further examined the cellular senescence-induced effects on apoptosis-related factors in myoblasts during differentiation. Our results documented that aged myoblasts exhibited a significant up-regulation of pro-apoptotic factors during myogenesis. More specifically, FoxO1 and p53 mRNA levels were significantly increased at the sixth day of differentiation, while FUCA expression was significantly higher on days twoand six of differentiation in aged myoblasts compared to controls (Figure 4A–C). Furthermore, we examined the protein expression levels of FoxO1 and p53 in control and senescent myoblasts during the differentiation process. Accordingly, the aged myoblasts showed increased levels of FoxO1 on the sixth day and of p53 on the sixth of their differentiation (Figure 4D,E). Moreover, we examined cell death in control and aged cells. We found that the cell viability of the aged myoblasts was decreased in contrast to the early and late apoptosis, which were significantly increased in the aged cells compared to control myoblasts (Figure 4F,G).

## 3. Discussion

This study examined the effects of myoblast aging on skeletal myogenesis in vitro, using a model of multiple cell divisions to replicate the cell senescence of C2C12 myoblasts. The senescent myoblasts exhibited an aging phenotype with G0/G1 cell cycle arrest, increased SA-β-gal activity and DNA damage, and increased percentages of early and late apoptotic cells, along with an increased expression of the aging-related proteins and cell cycle inhibitors p16 and p21 in their early differentiation. Moreover, our findings documented that, compared to control cells, the aged myoblasts showed a reduction in the expression of MRFs and metabolic/anabolic factors along with increased expression inapoptotic, atrophy and inflammatory factors, indicating a diminished differentiation capacity and a disrupted myogenic lineage of these senescent muscle cells.

Induction of cell senescence is typically associated with increased expression of aging-related proteins, such as p16 and p21, high levels of SA-β-galactosidase activity, extensive DNA damage, and elevated levels of apoptosis factors such as FoxO1 and p53 [18,19,55,56], which have been further associated with other cellular dysfunctions [57,58]. In this study, we characterized the effects of replicative senescence on cellular and molecular responses during myoblast differentiation.

Myogenic differentiation is driven by multiple signal transduction and mechanotransduction pathways which coordinate the balance between muscle growth and atrophy, or protein synthesis and protein degradation [27,59,60,61]. Our findings documented a delayed or reduced ability of aged myoblasts to differentiate, as demonstrated by the expression time course of the myogenic transcription factors (MRFs) and the morphological characteristics of these cells during the differentiation process. Apparently, the cell differentiation program of the aged myoblasts appeared to be late, although progressive, throughout the experimental period compared to the control cells, exhibiting a time-shift of the expression pattern of MRFs during myogenesis, although continuing to possess the myogenic differentiation potential. Interestingly, the delay in the myoblast differentiation seems to similarly affect the responses of both the early and late myogenic factors, implying a similar impact of myoblast senescence on the overall differentiation program and not a specific effect depending on the distinct roles of these factors in a particular stage of myogenesis.

Myogenic regulatory factors function as the main activators of skeletal muscle differentiation and regulate the expression of several genes that encode structural and regulatory muscle proteins, while these transcription factors have also been demonstrated to interact with certain growth factors, such asIGF-1 [34]. More specifically, myogenic differentiation is associated with the induction of anabolic processes, increased rate of protein synthesis, and ultimately, muscle cell growth [34]. IGF-1 is a major anabolic factor involved in myogenesis and a central therapeutic target for enhancing muscle function in aging [32], while the potentially differential role of its isoforms in aged muscles has been reported [62]. To the best of the authors’ knowledge, this is the first study investigating the distinct expression profiles of IGF-1 isoforms in replicative senescent myoblasts, in vitro. Our data revealed that while there were no differences between the control and aged myoblasts regarding the expression of the IGF-1Ea during the myogenic differentiation, the IGF-1Eb isoform exhibited a reduced expression in the aged myoblasts during the first days of differentiation. These findings further support the notion of a differential regulation and role of IGF-1 isoforms in muscle biology and possibly in the myogenic program of aged muscle cells. More specifically, the IGF-1Eb isoform is known to produce the Eb peptide and this post-translational processing product, as well as its human counterpart (Ec peptide), has been documented to act as an IGF-1R independent competence growth factor in several biological systems [37,38,63,64,65,66,67,68,69,70]. Previous studies have examined the potential impact and the possible mechanisms of action of IGF-1 isoform over-expression on muscle aging in vivo [62,69,71]; however, more studies are needed to mechanistically investigate the potentially distinct roles of the IGF-1 isoforms and particularly of IGF-1Eb in the aging of myoblasts.

Another metabolic factor that was downregulated in the aged cells throughout their myogenic differentiation was PPAR-γ. This is in line with previous reports that the level of PPAR-γ mRNA expression diminished significantly in the skeletal muscle of aged rats, and this reduced expression may affect the PPAR-γ-induced regulation of skeletal muscle insulin resistance in aged muscle cells [72,73]. More specifically, PPAR-γ activation in skeletal muscle can have an important protective effect on glucose homeostasis and insulin resistance, because muscle cells secrete functional adiponectin in a PPARγ-dependent manner, improving insulin sensitivity [53]. Our findings further support the notion that aging may be associated with diminished PPAR-γ expression in the aged skeletal muscle, affecting its insulin resistance [72]. Along with the characterization of the myogenic/anabolic profile, this study also investigated the expression responses of muscle atrophy genes during the differentiation program of the aged myoblasts. The detailed mechanisms by which those genes drive an atrophic phenotype remain to be fully elucidated; nevertheless, it is considered that myostatin is a negative regulator of myogenesis via the deregulation of MyoD and the inhibition of protein synthesis [31,74]. Similarly, MuRF1 plays a major role in controlling a variety of muscle cell catabolic processes [75], while Atrogin1 is negatively involved in myogenesis by degrading myogenin [76]. Our study revealed that similarly to the occurrence of an anti-anabolic phenotype in the differentiating aged myoblasts compared to control cells, they also exhibited upregulation of muscle atrophy genes during myogenesis. Overall, our findings suggest that the myogenic program of senescent myoblasts is accompanied by a less anabolic/more catabolic drive compared to control cells.

In addition, various pro-apoptotic factors are involved in and regulate myogenic differentiation. Specifically, p53 suppresses muscle differentiation at the myogenin step [46], while its downstream effectors appear to control an inflammatory, cytokine-mediated inhibition of myogenic differentiation [46], supporting the increasing evidence regarding the role of inflammation in impairing myogenesis [77]. Moreover, FoxO1 is a fate decider within the myogenic lineage as opposed to an inducer of the myogenic program [78], while both p53 and FoxO1 share similar biological functions in controlling the balance between cell death and survival [79]. FUCA has also been documented to inhibit cell growth and induce cell death [80]. These pro-apoptotic factors appear to interact with muscle atrophy factors, becauseFoxO1 regulates the expression of myostatin [81], whilst p53 and FUCA affect the Murf1 and Atrogin1 actions through the ubiquitin proteasome pathway [82].

Our model of replicative senescence of C2C12 myoblasts revealed a consistent up-regulation of pro-apoptotic factors, accompanied by changes in the expression of the key pro-inflammatory factor IL-6 in the aged cells compared to controls during myogenic differentiation. These findings indicate the increased activation of apoptotic responses in the senescent myoblasts compared to control cells during their differentiation program into myotubes.

The model used in this study has limitations, because extensive cell duplications cannot warrant cell senescence. Nevertheless, our further characterization of the potential aging phenotype of those cells, in terms of the senescence-associated cell cycle arrest, increased β-galactosidase activity, DNA damage, and expression of cellular senescence markers in the aged cells, revealed that this model exhibits some fundamental characteristics of cell aging.

## 4. Materials and Methods

### 4.1. Cell Culture

#### 4.1.1. C2C12 Cell Culture

The C2C12 cell line of mouse myoblasts was obtained from American Type Culture Collection (Manassas, VA, USA) and cultured as previously described [83]. Briefly, cells were grown in Dulbecco’s modified Eagle’s medium (DMEM) supplemented with 10% fetal bovine serum (FBS), plus 1% penicillin/streptomycin at 37 °C in a humidified atmosphere of 5% CO_2_ in air, while the medium was changed every other day. The C2C12 myoblasts were seeded onto 6-well culture plates and maintained in growth media (GM) until 70–80% confluent, then switched to a differentiation medium (2% horse serum, 1% of penicillin/streptomycin in DMEM), which was changed every other day.

#### 4.1.2. Myoblast Aging through Multiple Population Doublings of C2C12 Cells

To investigate skeletal muscle cell aging, we used and further characterized a model of multiple population doublings of C2C12 myoblasts, which was previously described as a potential model to investigate muscle cell senescence [84]. To further characterize the potential aging phenotype of those cells, in addition to the initial characterization of this published model, in this study we expanded the number of multiple doublings of myoblasts from 58 (in the original model) to 80 doublings in our cultures, and we subsequently characterized the model in terms of the senescence-associated (i) cell cycle arrest;(ii) increased β-galactosidase activity;(iii) DNA damage; and (iv) expression of cellular senescence markers in the aged cells. Original stock C2C12 cells were seeded at 1 × 10^6^ cells in T75 flasks in 10 mL of GM and incubated for 48 h until 80% confluent. Cells were then trypsinized and seeded onto new T75 flasks again at 1 × 10^6^ for 48 h or at 5 × 10^5^ cells for 72 h, with doubling time and cell numbers being recorded throughout, as previously described [84]. This cycle was repeated 20 times over 50 days, creating a stock of cells that had undergone the multiple population doublings, compared with the original stock that was retained in liquid nitrogen, had undergone no doublings, and was used as the controls in all experiments (i.e., the same parental cells that had produced their progeny were used as control relative to the multiple population doubling-induced aged cells). There was no observable cell death between cell expansions (determined by a trypan blue exclusion test of cell viability).

### 4.2. Cell Lysis and RNA Extraction

Both control and aged C2C12 cells were harvested at the 0, 2nd, and 6th day of differentiation, and cell extracts were obtained by cell lysis using NucleoZOL (Mecherey-Nagel, Duren, Germany). Total RNA was isolated from the lysates according to the manufacturer’s recommendations. The extracted RNA was dissolved in RNAase-free water (Invitrogen, Carlsbad, CA, USA) and the concentration and purity were determined spectrophotometrically (ThermoNanodrop 2000, Thermo Scientific™, Waltham, MA, USA) by absorption at 260 and 280 nm. The integrity of total RNA was confirmed by visual inspection of the electrophoretic pattern of 18S and 28S ribosomal RNA in ethidium bromide-stained 1% agarose gels under ultraviolet (UV) light. The total RNA samples were stored at –80 °C until further analyses for the determination of the mRNA levels of the genes of interest by reverse transcription and semi-quantitative real-time polymerase chain reaction (PCR) procedures.

### 4.3. Reverse Transcription and Real-Time PCR

Total RNA from each sample was used to produce single-stranded cDNA by reverse transcription using reverse transcriptase ProtoScript II (NEB, Ipswich, MA, USA), and the resultant cDNAs were utilized in real-time PCR. More specifically, for the reverse transcription, 1 μg of total RNA from each sample was mixed with random primers mix (300 ng/reaction), oligod(T)23VN (300 ng/reaction) and nuclease-free water in a total volume of 8 μL, heated at 65 °C for 5 min, and then placed on ice. Next, the samples were mixed with 10μL ProtoScript II Reaction Mix and 2 μL Protoscript II Enzyme mix and incubated consecutively at 25 °C for 5 min and at 45 °C for 1 h, according to manufacturer’s recommendations. At the final step of the reverse transcription, the samples were heated at 80 °C for 5 min, to inactivate the enzyme, and stored at −20 °C. Real-time PCR analyses were performed using the Bio-Rad 96-well iCycler thermal cycler (Bio-Rad iQ5 Real-Time PCR Detection System, Hercules, CA, USA) and Bio-Rad reagents (iQ™ SYBR Green Supermix). The primer set sequences used for the specific detection of IGF-1 isoforms (IGF-1Ea, IGF-1Eb), MRFs (Myf5, MyoD, myogenin, MRF4), atrophy (myostatin, MuRF1, Atrogin1), pro-apoptotic (FoxO1, FUCA, p53), inflammatory (IL-6), and metabolic (PPAR-γ) factors are shown in Table 1. To prevent the detection of genomic DNA, the primer sets were designed to lie within different exons while, particularly, each set of primers for the detection of the IGF-1 isoforms was specific to detect only one specific IGF-1 transcript. Each PCR reaction contained 50 ng of cDNA, 10 μL SYBR green master mix, 1μM of each primer, and nuclease-free water to a total volume of 20 μL. The real-time PCR parameters were the following: initial denaturation at 95 °C for 5 min followed by 40 cycles of 30 s at 95 °C, 30 s at 62 °C for annealing, and 30 s at 72 °C for extension. Transcript levels of the genes of interest were assessed by automatically calculating the threshold cycle (Ct) as the number of cycles at which the measured fluorescence exceeded the threshold for detection. To normalize the amount of total RNA present in each PCR reaction and the mRNA expression (relative quantification-dCt) of the genes of interest, glyceraldehyde 3-phosphate dehydrogenase (GAPDH) was used as a house-keeping gene (internal standard). Each sample was analyzed in duplicate, and the resulting data were averaged. A melting curve™ was also generated by the Bio-Rad iQ5 Real-Time PCR Detection System software after the final cycle for each experimental sample, by continuously monitoring the Bio-Rad SYBR fluorescence (Hercules, CA, USA) throughout the temperature ramp from 70 to 95 °C. The specificity of the primers for the corresponding transcript was also confirmed by the melting curve analysis of samples, where there was only one melting curve for each sample, and electrophoretic analysis of the real-time PCR products further verified the specificity of the transcript of each gene of interest. Control for specificity included cDNA-free reactions and template-free reactions.

### 4.4. Protein Extraction and Immunoblotting Analysis

Total proteins were extracted from C2C12myoblasts as previously described [64]. Briefly, C2C12 myoblasts were washed with ice-cold PBS before lysing in 150 μL of RIPA buffer (Cell signaling, Danvers, MA, USA) supplemented with protease and phosphatase inhibitor cocktails (Cell Signaling, Danvers, MA, USA). Lysates were incubated on ice under shaking for 20 min in order to ensure complete lysis of the cells, centrifuged at 15,000 rpm for 20 min at 4 °C, and the supernatants retained. Protein content was determined using a BCA protein assay kit (Thermo Scientific, Waltham, MA, USA). Samples were stored in aliquots at −80 °C until Western blot analysis, as previously described [38,64]. Briefly, equal amounts of protein extracts (50 µg) from C2C12 myoblasts were mixed with a loading buffer (Invitrogen, Carlsbad, CA, USA), denatured at 95°C for 5 min, subjected to sodium dodecyl sulfate-polyacrylamide gel electrophoresis (SDS-PAGE) (12% (*w/v*) separating gel and 4% (*w/v*) stacking gel)and vertically electrophoresed at 100 V for 3 h. They were then transferred to polyvinylidene fluoride (PVDF) membranes (Bio-Rad, Hercules, CA, USA) by 100 V for 3 h at 4 °C. Membranes were incubated with a blocking solution containing 5% bovine serum albumin (BSA) in Tris phosphate-buffered saline (TBS; 10 mMTris, pH 7.6; 100 mMNaCl) plus Tween (0.1%v/vTween 20) (TBS-T) at room temperature for 1 h. After three washes with TBS-T for 10min each, blots were incubated with the following primary antibodies overnight at 4 °C under gentle shaking: for the immunodetection of p16 (1:500 dilution; with 5% milk in TBS-T,ab51243;Abcam, Cambridge, UK);p21 (1:500dilution; with 5% milk in TBS-T,sc-817; Santa Cruz, Dallas, TX, USA);MyoD (1:1000 dilution; with 5% milk in TBS-T,sc-377460; Santa Cruz, Dallas, TX, USA);myogenin (1:1000 dilution; with 5% milk in TBS-T, ab1835,Abcam, Cambridge, UK);FoxO1 (1:1000 dilution; with 5% milk in TBS-T, #9464; Cell Signaling, Danvers, MA, USA); andp53 (1:5000 dilution; with 5% milk in TBS-T,sc-126; Santa Cruz Dallas, TX, USA). After overnight incubation and three washes with TBS-T, membranes were incubated with a horseradish peroxidase-conjugated secondary anti-rabbit IgG (goat anti-rabbit, 1:2000 dilution;sc-2004,Santa Cruz, Dallas, TX, USA) or anti-mouse IgG (goat anti-mouse, 1:2000 dilution; sc-2005,Santa Cruz, Dallas, TX, USA) in TBS-T containing 2.5% BSA, for 1 h at room temperature. The expected bands were visualized by exposure of the membranes to X-ray film after incubation with an enhanced chemiluminescent substrate for 3 min (ECL Supersignal west-pico, ThermoScientific Waltham, MA, USA). Glyceraldehyde 3-phosphate dehydrogenase (GAPDH) (1:2000 dilution with 5% milk in TBS-T, sc-47724; Santa Cruz, Dallas, TX, USA) was used as an internal standard to correct for potential variation in the protein loading and to normalize the protein measurements on the same immunoblot. Band intensity was then semi-quantified using ImageJ software.

### 4.5. Immunofluorescence

Myoblasts cultured on chamber slides were stained using an indirect immunofluorescence method. Cells were rinsed in PBS and fixed with ice-cold 4% formaldehyde for 10 min at room temperature. They were permeabilized with PBS plus 0.2% Triton X-100 (Sigma-Aldrich) for 10 min and were blocked with 10% goat serum in PBS for 1h at room temperature. They were then incubated with primary antibody mouse anti-myosin heavy chain (1:100, MAB4470 R&D, Minneapolis, MN, USA) overnight at 4 °C. After three washes with PBS, 5 min at room temperature, cells were incubated for 30 min with goat anti-mouse IgG conjugated to the fluorescent Alexa 488 dye (1:2000, ab150113, Abcam, Cambridge, UK). After three washes, samples were stained with DAPI (1 μg/mL) and viewed under a microscope (Olympus BX40; Olympus Corporation, Tokyo, Japan). Moreover, morphological analyses of myotubes of the C2C12 cells in both control and aged groups have been performed with the ImageJ software.

### 4.6. Flow Cytometry

#### 4.6.1. Fixation of Cells for Cell Cycle

Cell cycle analysis is a very common flow cytometry application. By using a DNA-specific stain, one can determine a DNA profile, e.g., find a percentage of the population in G1, S, and G2/M. C2C12 murine skeletal myoblasts were grown until 80–90% confluency. Myoblasts were trypsinized, washed three times with PBS, and fixed in methanol/acetone (4:1) for 30 min at 4 °C. The cells were further washed twice with PBS and incubated with RNase A (100 μg/mL) for 20 min at RT. Finally, they were incubated with 10% propidium iodide and the DNA content was measured by propidium iodide intensity by using a flow cytometer (PartecCyFlow, Görlitz, Germany). The cell cycle phases were analyzed by the FlowJo software.

#### 4.6.2. Fixation of Cells for Annexin—Propidium Iodide (PI)

Muscle cells were stained with FITC Annexin-V Apoptosis Detection Kit (R&D systems, Minneapolis, MN, USA) according to the manufacturer’s instructions. Briefly, cultured cells were collected, washed with cold PBS, and then stained with Annexin-V-FITC (0.25 µg/mL) and PI for 15 min at room temperature in the dark. The stained cells were then analyzed by flow cytometry, acquiring 1 × 10^5^ gated events according to a large gate established on cell forward and side scatters within 30 min from staining. Gated cells were separated into four quadrants: early apoptotic cells (Annexin-positive/PI-negative), necrotic cells (Annexin-negative/PI-positive), late apoptotic cells (Annexin-positive/PI-positive), and viable cells (Annexin-negative/PI-negative).

### 4.7. β-Galactosidase Staining (SA-β-gal)

C2C12 murine skeletal myoblasts were grown until 80–90% confluency. Following this, the cells were washed, fixed, and stained for β-galactosidase (SA-β-gal; a common marker for cell senescence), using the X-gal staining solution, then incubated overnight at 37 °C (as per the manufacturer’s instructions) (#9860 Cell Signaling, Danvers, MA, USA). The cells were then examined under a microscope at both 10× and 20× magnification (TE200; Nikon Instruments) for the development of the X-gal stain (blue). Images were captured (MetaMorph, version 5.0) with a camera at 10× and 20×.

### 4.8. Alkaline Single-Cell Gel Electrophoresis (Alkaline Comet Assay)

The single-cell gel electrophoresis assay was performed under alkaline conditions as previously described [85]. Myoblasts were cultured until 80% confluency in Dulbecco’s modified Eagle’s medium (DMEM) supplemented with 10% fetal bovine serum (FBS), plus 1% penicillin/streptomycin.C2C12 myoblasts were suspended in low melting point agarose (1%) in PBS at 37 °C, and spread onto specifically designed slides purchased from Trevigen (Comet Assay^®^ HT Slide, Trevigen, Gaithersburg, MD, USA). After lysis of cellular membranes at 4 °C for 2 h, slides were placed in a horizontal gel electrophoresis chamber and incubated in pre-chilled electrophoresis buffer for 40 min. Electrophoresis was performed for 30 min at 1 V/cm. Afterwards, slides were washed with neutralization buffer and distilled H_2_O and left to dry overnight. Gels were stained with SYBR Gold Nucleic Acid Gel Stain (Thermo Fischer Scientific, Waltham, Massachusetts, United States) and analyzed with a fluorescence microscope (Zeiss Axiophot, Oberkochen, Germany). Olive tail moments (OTM = (tail mean− head mean) × (% of DNA)/100) of at least 200 cells/treatment condition were evaluated. Comet parameters were analyzed with the ImageJ Analysis/Open Comet software. Experiments were performed in triplicate.

### 4.9. Statistical Analysis

One-way analysis of variance (ANOVA) with Dunn’s multiple comparison post hoc test or Student’s *t*-test was used for statistics, using GraphPad Prism 5. All experiments were performed in triplicate and data are presented as the mean ± standard error of the mean (S.E.M). The level of statistical significance was set at *p* < 0.05.

## 5. Conclusions

The aging-related progressive loss of skeletal muscle mass, known as sarcopenia, is the most common type of muscle atrophy and is associated with significant impairment of muscle function. Aging is a very complex biological phenomenon, and our understanding of skeletal muscle aging is limited. In vitro models of muscle cell senescence can greatly contribute to the improvement of our knowledge of cellular and molecular aspects of aging-induced muscle changes. This study characterized a model of replicative myoblast senescence and demonstrated that a diminished myogenic/anabolic potential characterizes the aged myoblasts which, in combination with the induction of apoptotic and muscle atrophy factors, indicates a disrupted myogenic lineage in the senescent muscle cells. These findings might serve as a molecular signature of myogenesis in replicative senescent myoblasts and a valuable resource for developing more focused in vitro experimental designs to further characterize the cellular and molecular mechanisms of skeletal muscle alterations in aging.

## Figures and Tables

**Table 1:** The sequences of the specific sets of primers used for RT-PCR analyses.

Target Gene	5′-3′ Forward Primer Sequence	5′-3′ Reverse Primer Sequence	Product Length
***GAPDH***	CAA CTC CCT CAA GAT TGT CAG CAA	GGC ATG GAC TGT GGT CAT GA	118
***Myf5***	CTA TTA CAG CCT GCC GGG AC	CTC GGA TGG CTC TGT AGA CG	232
***MyoD***	TGC TCC TTT GAG ACA GCA GA	AGT AGG GAA GTG TGC GTG CT	141
***Myogenin***	AGG AGA GAA AGA TGG AGT CCA GAG	TAA CAA AAG AAG TCA CCC CAA GAG	430
***MRF4***	AGG GCT CTC CTT TGT ATC CAG	TGG AAG AAA GGC GCT GAA GA	579
***IGF-1Ea***	GTG GAC GCT CTT CAG TTC GT	GCT TCC TTT TCT TGT GTG TCG ATA G	262
***IGF-1Eb***	GTC CCC AGC ACA CAT CGC G	TCT TTT GTG CAA AAT AAG GCG TA	259
***FUCA***	TTT GGT CGG TGA GTT GGG AG	CCA TTC CAA GAG CGA GTG GT	76
***FoxO1***	AGT GGA TGG TGA AGA GCG TG	GAA GGG ACA GAT TGT GGC GA	96
***p53***	GAG AGA CCG CCG TAC AGA AG	AGC AGT TTG GGC TTT CCT CC	317
***Myostatin***	CTG TAA CCT TCC CAG GAC CA	GCA GTC AAG CCC AAA GTC TC	104
***MuRF1***	AGG GCT CCC CAC CAC CTG TGT	TGC CCT CTC TAG GCC ACC G	310
***Atrogin1/*** ***MAFbx***	AAC AAG GAG GTA TAC AGT AAG G	AAT TGT TCA TGA AGT TCT TTT G	322
***IL-6***	CTA TGA ACT CCT TCT CCA CAA GCG CCT T	GGG GCG GCT ACA TCT TTG GAA TCT T	301
***PPAR-γ***	GTT CAT GCT TGT GAA GGA TGC	ACT CTG GAT TCA GCT GGT CG	359

## Data Availability

Not applicable.
